# Direct Synthesis and Morphological Characterization of Gold-Dendrimer Nanocomposites Prepared Using PAMAM Succinamic Acid Dendrimers: Preliminary Study of the Calcification Potential

**DOI:** 10.1155/2014/103462

**Published:** 2014-01-28

**Authors:** E. Vasile, A. Serafim, D. Petre, D. Giol, P. Dubruel, H. Iovu, I. C. Stancu

**Affiliations:** ^1^METAV Research and Development, 31 C.A. Rosetti, Sector 2, 020015 Bucharest, Romania; ^2^Advanced Polymer Materials Group, University Politehnica of Bucharest, 149 Calea Victoriei, Sector 1, 010072 Bucharest, Romania; ^3^Polymer Chemistry and Biomaterials Group, Ghent University, Krijgslaan 281 (S4), 9000 Ghent, Belgium

## Abstract

Gold-dendrimer nanocomposites were obtained for the first time by a simple colloidal approach based on the use of polyamidoamine dendrimers with succinamic acid terminal groups and dodecanediamine core. Spherical and highly crystalline nanoparticles with dimensions between 3 nm and 60 nm, and size-polydispersity depending on the synthesis conditions, have been generated. The influence of the stoichiometric ratio and the structural and architectural features of the dendrimers on the properties of the nanocomposites has been described. The self-assembling behaviour of these materials produces gold-dendrimer nanostructured porous networks with variable density, porosity, and composition. The investigations of the reaction systems, by TEM, at two postsynthesis moments, allowed to preliminary establish the control over the properties of the nanocomposite products. Furthermore, this study allowed better understanding of the mechanism of nanocomposite generation. Impressively, in the early stages of the synthesis, the organization of gold inside the dendrimer molecules has been evidenced by micrographs. Growth and ripening mechanisms further lead to nanoparticles with typical characteristics. The potential of such nanocomposite particles to induce calcification when coating a polymer substrate was also investigated.

## 1. Introduction

As we are contemplating the extraordinary scientific and technological progress, “bio-” and “nano-” became nowadays common prefixes characterizing high-tech materials and devices. The interest and awareness of scientists of the use of gold and silver nanoparticles in various applications including biomedical uses have increased tremendously in the last ten years [1–11].

Obtaining noble metals nanoparticles in the presence of dendrimers represents nowadays a common procedure [1, 11–22]. However, the concept of silver- and gold-dendrimer nanocomposites is not new; it was developed approximately 17 years ago and it was based on the immobilization of preorganized metal ions in dendrimer hosts [12]. Dendrimers are well-known globular macromolecules presenting a typical tree-like architecture divergent from a central core and defined yet variable multifunctionality. They are interesting templates and reactive containers for noble metal precursors, able to initiate and direct the growth of the metallic nanometric crystals within a framework. Nevertheless, they act as stabilizers and/or encapsulating hosts. The main roles played by dendrimers, according to the original “dendrimer nanocomposite concept” introduced through the pioneering work done by Zawodzinki, Balogh, Tomalia, and Crooks, are as follows: (1) preorganization of ions involving guest-host interactions and (2) nanoreactor for *in situ* reduction reactions [12]. Furthermore, different morphological features such as shape, size, and size-distribution can be controlled through a rational combination of reagents and reaction conditions. Different parameters such as the dendrimer type (including internal structure and external functionality), the generation number, and the stoichiometry are of paramount importance when aiming at the control of the properties of the dendrimer-metal nanocomposites. PAMAM (poly(amidoamine)) and PPI (poly(propyleneimine)) dendrimers are attractive candidates as nanoreactors and stabilizers for metallic nanoparticles [18, 22, 23]. It was shown that the variable multifunctionality and intrinsic architecture with internal cages, whose complexity is increasing with the generation number, strongly affect the autoreduction capacity, as well as the size, morphology, and aggregation/stability of the generated and/or stabilized nanoparticles [1, 14–18, 23–26]. Different PAMAM molecules were used in these studies, and the effects of their generation number, concentration, and external functionality over the morphostructural features and aggregation behaviour of the metal nanoparticles have been established [14, 27]. Gold or silver nanocomposites were mainly prepared using PAMAM with ethylenediamine (EDA) core [12, 27] and amine [12, 14, 16, 18, 28], carboxylate [12, 14], thiol [27], and hydroxyl [14] terminal functional groups. The preparation of dendrimer-stabilized NPs by simply mixing alcohol terminated dendrimers or dendrimers with tertiary amine groups with AuCl_4_ has been reported [29–32].

In this work, we report for the first time the formation and characterization of gold-dendrimer composite nanoparticles using PAMAM succinamic acid (PAMAM-SA) dendrimers with 1,12-dodecanediamine core. These dendrimers were selected due to their multicarboxylic functional shell. Such macromolecules are extremely appealing when targeting the induction of calcification phenomenon by a biomimetic approach. Our aim was to generate novel composite nanoparticles with –COOH end functions and to investigate their potential as nanometric platforms capable to form biomimetic hydroxyapatite (HA) when used as coating of biomaterials incubated in synthetic body fluids (SBF). Accordingly, we first synthesized the –COOH reach gold-PAMAM-SA nanocomposites and then we investigated their structure and morphology. In a second step, we used selected nanocomposite particles to coat a biocompatible polymer, poly(2-hydroxyethyl methacrylate) (PHEMA), known to not promote biomineralization in SBF under physiological conditions. To the best of the authors' knowledge, PAMAM-SA dendrimers have never been used by other groups to generate gold nanoparticles nor to induce calcification. Accordingly, we have investigated the formation of such gold-dendrimer nanoparticles and the effect of dendrimers on the morphology, structure, and “afterlife” characteristics of the nanocomposite. In this study, a special interest was devoted on one hand to the relationship between the stoichiometry and the preorganization of the metal precursors and, on the other hand, to the “afterlife” interactions and assembling properties. The influence of the concentration and of the generation number of the dendrimers on the properties of the so-formed nanocomposites was studied through various methods. In addition, this study continues our interest in the design of self-calcifying biomaterials for bone engineering [2, 33]. HA is recognized for its biological affinity to natural bone [34]. Therefore, HA coatings are extensively used to improve the integration of bone implants and the interaction at the interface bone scaffold. We recently proved the potential of COOH-functionalized gold nanoparticles to induce the biomimetic mineralization of PHEMA for bone regeneration aim [2]. Our interest in the eventual calcification potential of the carboxylic shell of PAMAM-SA dendrimers is justified by the importance given to some bone proteins containing anions (carboxyl and phosphates) in the induction of the mineralization phenomenon. It became a common procedure nowadays to mimic the chemistry of these proteins using anionic groups (carboxyl, phosphate, and sulfonic) in the design of bone biomaterials [2, 33–38]. On the other hand, attempts to induce the so-called *template mineralization* on a multifunctional support with defined architecture and dimensions are reported [39–42]. Furthermore, the design of bone biomaterials with topographical features enhancing osteoblasts adhesion and activity is another recent trend in the development of bone biomaterials [43–47]. Therefore, the development and investigation of gold-dendrimer nanocomposites as coating of PHEMA were considered interesting for bone regeneration. The calcification potential of such materials was explored in SBF1.5x.

## 2. Materials and Methods

### 2.1. Materials

For nanocomposite synthesis, gold (III) chloride hydrate (or chloroauric acid) (HAuCl_4_ *·*H_2_O) (Sigma-Aldrich, Steinheim, Germany) was used as precursor for the obtaining of gold nanoparticles. Polyamidoamine dendrimers PAMAM-succinamic acid (PAMAM-SA) with 1,12-diaminododecane core, generations 2, 4, and 6, respectively, were used as received from Sigma-Aldrich (10% aqueous solutions). All other chemicals were from Sigma-Aldrich and used as received. The water was purified with a GFL 2101–2108 bidistiller apparatus.

For SBF, sodium chloride (NaCl), sodium hydrogen carbonate (NaHCO3), potassium chloride (KCl), dipotassium hydrogen phosphate trihydrate (K2HPO4·3H2O), magnesium chloride hexahydrate (MgCl2*·*6H2O), calcium chloride (CaCl2), and sodium sulfate (Na2SO4) were used. All salts were supplied from Sigma-Aldrich and used as such. Hydrochloric acid (1 N) was purchased from CHIMOPAR Bucharest. Tris (tris-hydroxymethyl aminomethane) 99+% was from Sigma-Aldrich. PHEMA cylinders were prepared as reported before [2].

### 2.2. Techniques

#### 2.2.1. Preparation of Metal-Dendrimer Nanoparticles

NPs were prepared without additional reducing agents and at room temperature.

The ratio Au (III)/tertiary amine groups from the external functional shell of the dendrimers was kept constant; two such ratios were used: 5 and 2.5.

For example, for a molar ratio Au (III)/tertiary amines from PAMAM-SA-G6 of 5 to 2 mL aqueous solution of HAuCl_4_ (5 mM), the corresponding amount of PAMAM-SA-G6 aqueous solution was added under vigorous stirring. The yellow colour of the precursor solution vanished immediately after the addition of the dendrimer. This indicates the rapid complexation between the functional groups of PAMAM-SA and gold. Approximately 20–30 minutes later, the slightly yellow colour of the mixture turns light blue and then purple. Homogenisation was continued at room temperature for 48 hours. The colour turns deep purple. Agglomerations become visible after 24 hours.

By adjusting the ratio, metal/tertiary amine different NPs were obtained with the three types of dendrimers: PAMAM-SA-G2, PAMAM-SA-G4, and PAMAM-SA-G6. The two classes of resulting products were further denoted Au-Gi-5, Au-Gi-2.5, Au stating for atomic gold, Gi for the generation of the dendrimer and 5 and, respectively, 2.5 for the molar ratio between Au(III) and tertiary amines.

In addition, to better understand the influence of the dendrimer concentration over the formation and morphology of gold NPs, as well as the stabilization efficiency of this dendrimer, different molar ratios were used between Au(III) and PAMAM-SA-G6: 10 : 1, 5 : 1, 2.5 : 1, and 1 : 1 Au (III): tertiary amines groups, respectively. The resulting NPs denoted Au-G6-10, Au-G6-5, Au-G6-2.5, and Au-G6-1.

#### 2.2.2. Characterization

*Fourier Transform Infrared Spectrometry (FTIR).* FTIR spectra were collected using spectrometer, JASCO 4200, equipped with a Specac Golden Gate attenuated total reflectance (ATR) device, using a resolution of 4 cm^−1^ and an accumulation of 60 spectra in the 4000–600 cm^−1^ wavenumbers interval. Dendrimers were used as control samples. A drop of colloidal solution or dendrimer solution was placed onto the crystal and allowed to evaporate in order to obtain a thin coating that was further analyzed.

*UV-Vis Spectroscopy. *Optical properties were disclosed through *UV-Vis spectra* recorded using a CINTRA 101 spectrometer. Quartz cells with 1 cm pathway have been used.

*Morphological and structural information* with respect to the shape, size, and eventual assembling or agglomeration of the formed gold-dendrimers NPs is obtained by different microscopic techniques. *Scanning electron microscopy (SEM)* was used to assess general aspect of agglomerated NPs deposited onto a glass support. The study was performed using a QUANTA INSPECT F SEM device equipped with a field emission gun (FEG) with 1.2 nm resolution and with an X-ray energy dispersive spectrometer (EDS). *Transmission electron microscopy (TEM)* and high resolution TEM (HR-TEM) analyses (TECNAI F30 G^2^ S-TWIN microscope operated at 300 kV with EDX and EELS facilities) were used for detailed analysis of morphological features and crystallinity. 20 *μ*L of aqueous-ethanolic solution containing dispersed NPs was dropped directly onto the C-coated cupper grid and allowed to dry in air for 30 minutes before the measurement. The size-distribution histogram was obtained using 200 nanoparticles that are randomly selected. *Atomic force microscopy (AFM)* was performed on precipitated NPs on glass slides. The roughness was investigated using a multimode scanning microscope (Digital Instruments, USA), equipped with a Nanoscope IIIa controller. Different scan sizes were recorded (1 *μ*m × 1 *μ*m and 50 *μ*m × 50 *μ*m) using a silicon cantilever (OTESPA, Veeco) in “tapping” mode in air; nanoscope software version was used.

#### 2.2.3. Calcification Study

The incubation solution SBF 1.5x was prepared modifying a recently reported protocol [2]. The acellular solution is 1.5-fold more concentrated than the common SBF. SBF has very similar ionic composition with the human plasma (with ion concentrations in mM: Na^+^ 142.0, K^+^ 5.0, Mg^2+^ 1.5, Ca^2+^ 2.5, Cl^−^ 147.8, HCO_3_^−^ 4.2, HPO_4_^2−^ 1.0, and SO_4_^2−^ 0.5).

Au-G6-1 nanocomposites were generated onto the surface of PHEMA cylinders. The synthesis was performed as described above in the presence of the polymer substrate. After 24 hours, the polymer cylinders were removed from the reaction vessel and immersed for 48 hours in ddw to remove eventually nonbound gold-dendrimer nanocomposites from the surface. Then, they were soaked in 10 mL of SBF, at 37°C, for 7 days. During the test, the media were renewed every 24 hours. After incubation, the samples were gently rinsed with distilled water to remove the residual salts physically deposited onto the samples and then dried at 40°C. The success of the biomineralization was explored through ATR FTIR and TEM (and HR-TEM) with EDX. For TEM and HR-TEM analyses, a thin layer was removed by scratching the surface of the polymer substrate after the incubation in SBF 1.5x (PHEMA is a glassy material when dried) and deposited on a TEM copper grid covered with a thin amorphous carbon film with holes.

## 3. Results and Discussion

**Scheme 1:**
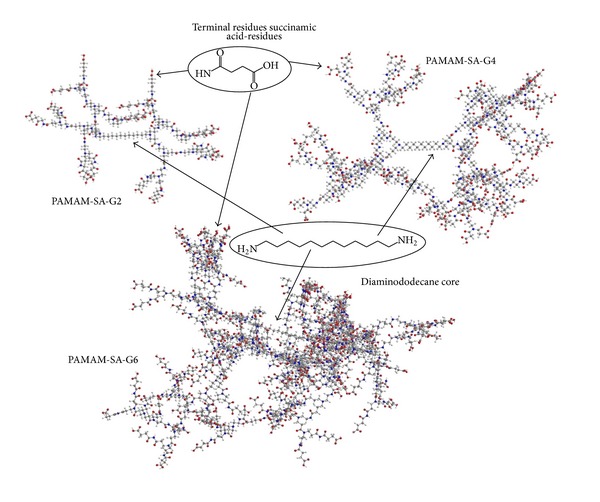
Schematic view of the three types of dendrimer molecules used in this study: PAMAM-SA-G2, PAMAM-SA-G4, and PAMAM-SA-G6.

PAMAM-SA dendrimers selected for this study have a longer core unit consisting in 1,12-dodecanediamine, when compared to the short EDA, while the structural units of the dendrimer branches remain [–CH_2_–CH_2_–CO–NH–CH_2_–CH_2_–N–] (Scheme 1). Due to the longer core unit, PAMAM-SA molecules used in this work are more open structures, with larger central cages, when compared to PAMAM dendrimers used in the synthesis of metal nanoparticles by other groups. In the present work, we investigated (1) the synthesis of gold nanoparticles in the presence of PAMAM-SA dendrimers and in the absence of additional reducing agents at room temperature, (2) the influence of the generation number and concentration of dendrimers over the structure, size, and morphology of the resulting composite nanoparticles, (3) the crystallinity of the formed particles, and (4) the postsynthesis life of the particles, in terms of stability and aggregation.

### 3.1. Generation of Crystalline Composite Nanoparticles in the Presence of PAMAM-SA

The first part of this study was devoted to the investigation of the potential of PAMAM-SA with 1,12-dodecanediamine core to induce the formation of gold nanoparticles in the absence of additional reducing agents. Three types of macromolecules, with generation numbers 2, 4, and 6, respectively, have been utilized. Two ratios between the gold precursor and the terminal groups of the dendrimers have been used: 2.5 and 5. After approximately 15 minutes from the moment the two solutions were put into contact, the colour of the reaction mixture significantly changed. The appearance of a deep red colour in all the reaction media was a representative piece of evidence for the successful synthesis of gold nanoparticles. The confirmation of this observation was obtained by means of UV-Vis spectra; the signals specific for the surface plasmon resonance (SPR) of gold were identified as broad bands with maxima in the interval from 525 to 535 nm. The broad SPR signals are representative for low dimension nanoparticles. These measurements indicated that, similarly to other classes of dendrimers, the generation numbers as well as the concentration of the dendrimers play key roles in the characteristics of the generated nanoparticles. A hypsochromic shift of the SPR signals was recorded owing to the influence of the increasing concentration of dendrimers in the reaction medium; this is assigned to diminished particles size of the formed nanoparticles. This observation is in agreement with other studies reporting on the obtaining of smaller gold nanoparticles when using increased concentration of PPI dendrimers [25, 26] or PAMAM with EDA core units and various terminal groups [18, 27].

**Figure 1:**
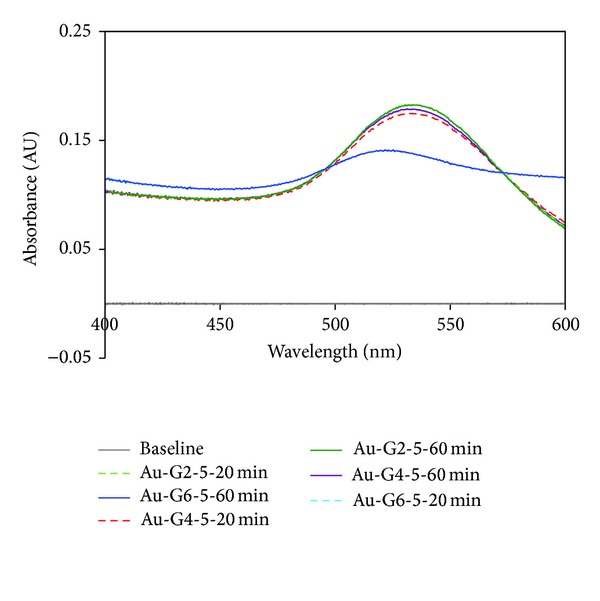
Hypsochromic shift of the SPR signal of gold nanoparticles Au-Gi-5, due to increasing the generation of dendrimers from G2 to G6. The spectra recorded after 20 minutes and after 60 minutes, respectively, (from the moment when the dendrimers and gold precursors were mixed) show good stability of the nanoparticles in the first reaction hour.

Increasing the generation of the dendrimer from G2 to G6 is also associated with a blue shift of the SPR maxima; this phenomenon is attributed to the formation of smaller nanoparticles (Figure 1). This is also a typical UV-Vis feature for dendrimer-assisted synthesis of metal nanoparticles. Furthermore, it was observed that increasing the generation number of the dendrimer from 2 to 6 leads to broader SPR signals. Such wavelength broadening would correspond, according to the results reported by Kiang [48], to the formation of self-assembled clusters.

**Figure 2:**
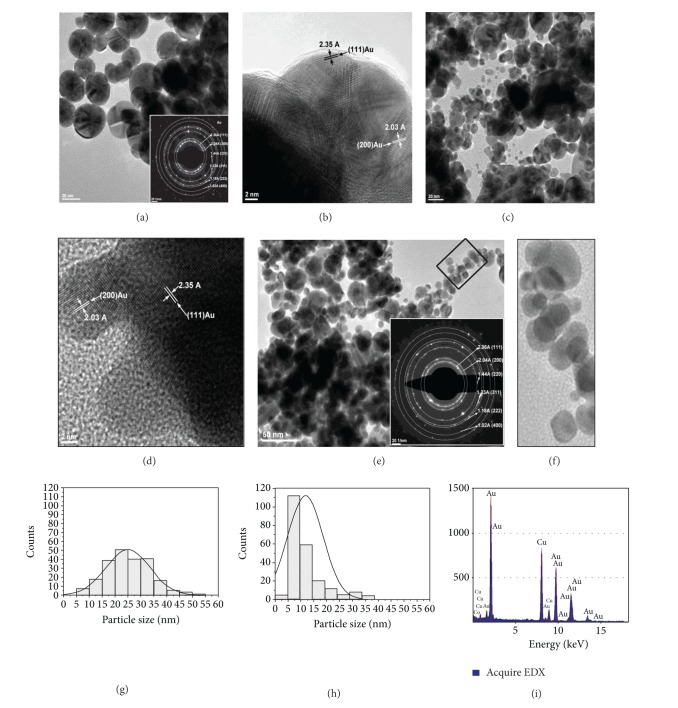
TEM and HR-TEM images presenting the influence of dendrimer concentration over the dimension of Au-G2-5 ((a) and (b); scalebar 20 nm) and Au-G2-2.5 ((c)–(e); scalebar 2 nm). (a), (c), (e) General views with insets presenting representative SAED patterns for gold (fcc); (b) two Au-G2-5 highly crystalline nanoparticles coated by a thin organic exterior shell; (d) two small but still crystalline Au-G2-2.5 nanoparticles onto the surface of a larger particle; (e) different populations of particles coexist; the small particles are more numerous; spherical entities around 3.8 nm are visible; (f) selected area from (e). Corresponding histograms of the particles size distribution are also included ((g), (h)). (i) EDX spectrum as recorded for Au-G2-2.5.

This assumption as well as the size and morphological investigation of the metal nanoparticles was further verified by means of TEM and HR-TEM analyses. The obtained results confirmed the above described dependency between the dendrimer concentration and the size of the NPs. More specifically, when the concentration of the dendrimer is doubled, TEM analyses indicated a general decrease trend in the size of the nanoparticles. Accordingly, all the synthesized Au-Gi-2.5 nanoparticles are larger when compared to their corresponding Au-Gi-5.  Figure 2 is representative with this respect and it shows the microscopic appearance of the two types of gold-dendrimer nanocomposites synthesized using PAMAM-SA-G2. It can be observed that Au-G2-5 nanoparticles are spherical crystalline structures, with mean dimensions of 25.01 ± 8.49 nm, while Au-G2-2.5 nanocomposites present average dimensions of 12.22 ± 7.16 nm (as obtained from the size-distribution histograms). This dimensional decrease is also accompanied by a modification of the size polydispersity. Accordingly, when lower concentration of G2 was used, the obtained Au-G2-5 particles were more homogeneous in size, with a dominant population with dimensions between 15 and 35 nm, respectively, and lower number of small particles (minimum 6.5 nm) and big particles (maximum 54.6 nm). On the other hand, when the concentration of PAMAM-SA-G2 was increased twice, the polydispersity is narrower, with a dominant population ranging between 5 and 10 nm, followed by a second population with diameters from 10 and 15 nm; lower number of Au-G2-2.5 nanoparticles with detected minimum of 3.8 nm and detected maximum of 28.3 nm were also identified in the system. This behavior is further explained. PAMAM-SA-G2 has a total number of 14 tertiary amines known to efficiently reduce Au(III) to Au^0^. Accordingly, the intradendritic space would initially contain metal agglomerations due to preorganization phenomenon; then nucleation areas appear and, during the growth phase, the space is filled with Au^0^. The growth of the crystalline gold inside the dendrimer host may continue until PAMAM-SA-G2 is encapsulated into the nanocomposite particles serving as both nanoreactor and internal templates. Increasing the concentration of dendrimer while keeping the same amount of precursor Au(III) corresponds to providing a double number of hosting templates accumulating Au(III), subsequently allowing the reduction to Au^0^ by the tertiary amines leading to local accumulation of the metal inside the dendrimer cages. Thereafter, gold crystals nucleate and further grow leading to a higher number of polycrystalline nanocomposites with smaller dimension when compared to Au-G2-5. Larger particles form only accidentally when the growth of the metal continued or particle ripening at room temperature occurred. Another very important finding is that, in the two studied situations, the nanocomposites present dimensions above the dendrimer size. This is not necessarily an intriguing aspect since PAMAM-SA-G2, as stated before, has an open structure with short divergent branches allowing the growth of the crystalline Au-G2 nanocomposites with the encapsulation of the dendrimer. HR-TEM micrographs also revealed the presence of a thin organic layer at the surface of some particles, as shown in Figure 2(b).

**Figure 3:**
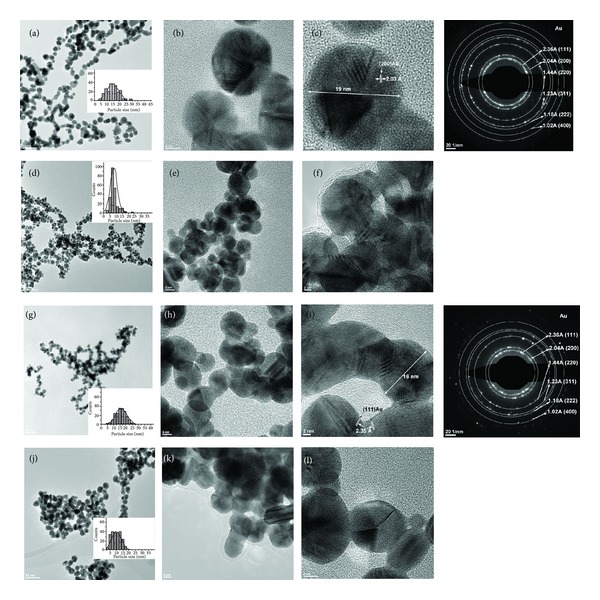
TEM and HR-TEM micrographs of (a)–(c): Au-G4-5; (d)–(f): Au-G4-2.5; (g)–(i): Au-G6-5; (j)–(l): Au-G6-2.5 nanocomposites after 48 hours. Specific size-distribution histograms were included. SAED results are representative for gold (fcc) polycrystalline structures.

Increasing the dendrimer generation number to 4 and 6, respectively, modifies the structural and morphological features of the resulting Au-G4 and Au-G6 nanocomposites. Definitely, smaller particles are obtained when a dendrimer with a higher generation number is used (Figure 3). These two types of dendrimers are more branched macromolecules when compared to PAMAM-SA-G2; they already present defined internal cavities capable of hosting Au(III). PAMAM-SA-G6 already has a globular structure with 256 terminal groups onto its shell. Furthermore, PAMAM-SA-G4 has 62 tertiary amines while PAMAM-SA-G6 presents 254 tertiary amines.

The architectural features, such as branches length and internal porosity of the dendrimers, initially increase the loading efficiency of the dendrimer nanoreactors with Au(III). Then, the structural characteristics act on the reduction of Au(III) to Au^0^. Figure 3 is representative with this respect. It describes the morphological features of Au-G4 and Au-G6 nanocomposites. The influence of the generation number and concentration is in perfect agreement with the previously described UV-Vis data. As general behavior, increased concentration of dendrimers in the reaction mixture diminishes the dimension of the generated particles. Accordingly, the average dimension of Au-G4-5 is 14.64 ± 4.58 nm (Figures 3(a)–3(c)), while that of Au-G4-2.5 is 7.99 ± 2.82 nm (Figures 3(d)–3(f)). The same applies for Au-G6 nanocomposites which are smaller than the corresponding Au-G4. The mean size of Au-G6-5 is 15.93 ± 4.44 nm (Figures 3(g)–3(i)), while that of Au-G6-2.5 is only 9.79 ± 3.56 nm (Figures 3(j)–3(l)). Visibly narrower distribution of the size-dependent populations of Au-G4-2.5 particles is evident when compared to Au-G4-5. Interestingly, the modification of the polydispersity of Au-G6-5 with respect to Au-G6-2.5 particles is less significant than the change specific to Au-G4 materials. When the concentration of the dendrimers was increased two times, the dominant populations have dimensions ranging from 5 to 15 nm, when compared to 10–22 nm in Au-G6-5. This could be explained through the higher stabilization efficiency of PAMAM-SA-G6. Actually, the composition, the intrinsic 3D architecture, and the internal organization of higher generation dendrimers have a synergistic influence onto their capacity of acting as (1) host (through the large internal cages), (2) nanoreactor (high number of tertiary amines acting as reducing sites for Au(III)), (3) 3D template for the development of the polycrystalline structure, and (4) stabilizing agent (through the external functional shell). All the above mentioned phenomena subscribe the combination of mechanisms described by Balogh et al. since 1999 [12]. According to this work, gold precursor penetrates the dendrimer and its distribution inside the host is homogeneous due to isotropic diffusion [12]. From a stoichiometric point of view, 62 gold atoms and, respectively, 254 gold atoms per molecule can be theoretically immobilized by PAMAM-SA-G4 and PAMAM-SA-G6, respectively. Starting from this analysis, it is easy to imagine the complexity of the simultaneous and competitive phenomena occurring in the dendrimer nanoreactors.

Furthermore, all the generated nanoparticles are polycrystalline structures, with visible atomic planes indicating gold face-centered cubic (fcc) lattice as proved by HR-TEM and SAED. Typical spacing for adjacent lattice planes of (fcc) gold has been detected as shown in Figures 2 and 3: 2.36 Å corresponding to (111) planes, 2.04 Å corresponding for (200) planes, 1.44 Å corresponding to (220) planes, 1.23 Å corresponding to (311) planes, 1.18 Å corresponding to (220) planes, and 1.02 Å corresponding to (400) planes.

**Figure 4:**
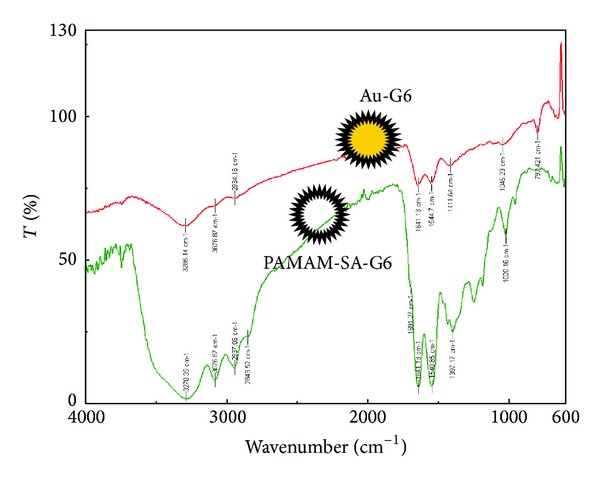
FTIR spectra of gold nanocomposites formed using PAMAM-SA-G6 dendrimers; spectra recorded after extensive rinsing with ddw. PAMAM-SA-G6 was used as control.

EDX spectra have been collected in order to verify the composition of each type of particles. The EDX spectrum of *Au-G2-2.5 *is presented in Figure 2. It indicates the presence of gold. FTIR spectra of the Au-Gi particles revealed the presence of the dendrimer in all the analyzed structures. The dendrimers have been used as positive controls. As an example, the FTIR spectrometric features of Au-G6-2.5 particles presented typical vibrations characteristics to the corresponding dendrimer. PAMAM-SA-G6 presents broad vibration with a maximum at 3278 cm^−1^ characteristic to O–H, N–H signals at 3076 cm^−1^, C–H stretching vibrations at 2937 and 2843 cm^−1^, respectively, amide I and amide II peaks at 1641 cm^−1^ and 1540 cm^−1^, and a shoulder at 1691 cm^−1^ corresponding to the carbonyl vibration of carboxylic acid origin (Figure 4). Au-G6-2.5 particles present a broad vibration with maximum at 3285 cm^−1^ assigned to O–H stretching, N–H at 3076 cm^−1^, C–H symmetrical and asymmetrical stretching at 2934 cm^−1^ and 2840 cm^−1^, and amide I and amide II at 1641 cm^−1^ and 1544 cm^−1^, respectively. This is a strong piece of evidence confirming the gold-dendrimer nanocomposite nature of the crystalline particles.

### 3.2. Au-Gi as Nanostructured Porous Networks or Coatings

**Figure 5:**
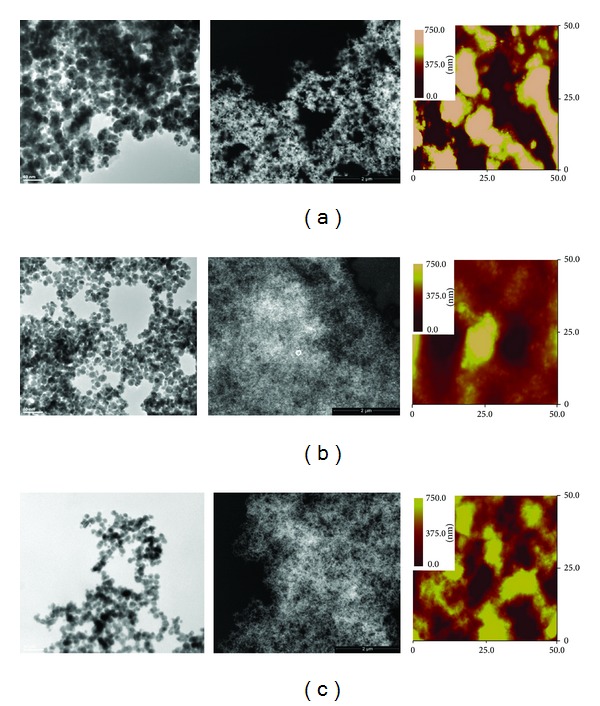
Morphology evaluation through TEM, SEM (50000x), and AFM (50 *μ*m × 50 *μ*m) of samples (a): Au-G2-5, (b): Au-G4-5, and (c): Au-G6-5 after two weeks after synthesis. Spherical particles are visible in TEM, with dimensions decreasing with the increasing generation of dendrimer. SEM reveals porous networks consisting in self-assembled nanoparticles. Nanostructured chains are entangled and delimitate internal pores. AFM confirms the results from TEM and SEM.

An interesting finding of our study regards the capacity of the generated nanocomposites to form nanostructured coatings when deposited onto solid surfaces. SEM and AFM imagings were used as techniques complementary to TEM to investigate the appearance of such coatings at two weeks after the synthesis. They revealed a perfect match with the observations already made by TEM analysis. Actually, all the synthesized gold-dendrimer nanocomposites present a high capacity of generating porous nanocrystalline structures, with porosity resulting from the assembled particles often displaying submicronic and even nanometric sized internal channels.  Figure 5 is representative with this respect. SEM images in Figure 5 are suggesting a decreasing size of the nanocomposites with increasing dendrimer generation number. This is also associated with denser nanocomposites porous layers. AFM results were in agreement with the SEM and TEM conclusions. It is generally recognized that physical interactions occur between dendrimer molecules leading to macromolecular aggregates in aqueous solutions. We speculate that, even despite vigorous ultrasonication before and stirring during synthesis, such dendrimer clusters exist and complicate even more the formation of the nanocomposites. In addition to this, the occurrence of Ostwald ripening processes after the synthesis may lead to the formation of more complex aggregates. We conclude that all these synergistic phenomena enhance the use of such synthesis procedure to generate nanostructured polycrystalline porous networks to be further used as such or as coatings.

### 3.3. Investigation of “Afterlife” Interactions

**Scheme 2:**
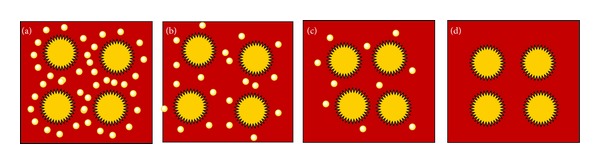
Schematic view of an ideal distribution of gold atoms (yellow circles) in the reaction mixture: (a) Au-G6-10, (b) Au-G6-5, (c) Au-G6-2.5, (d) Au-G6-1.

The second part of this study investigated the influence of the concentration of PAMAM-SA-G6 on the size and morphology of the so-formed gold-based nanocomposites. Four different concentrations have been used and the resulted nanocomposites have been denoted Au-G6-10 (corresponding to 10 Au(III): 1 tertiary amine), Au-G6-5 (corresponding to 5 Au(III): 1 tertiary amine), Au-G6-2.5 (corresponding to 2.5 Au(III): 1 tertiary amine), and Au-G6-1 (corresponding to 1 Au(III): 1 tertiary amine). We decided to investigate three situations where gold precursors were used in excess with respect to the reducing species and 1 with a 1 : 1 molar ratio. Even if the stoichiometry of the reduction of gold seems well defined by these ratios, the use of high generation dendrimers complicates the situation due to steric hindrance. More specifically, Scheme 2 simplistically presents an ideal situation when all the internal tertiary amines would allow the immobilization of one gold atom inside the dendrimer shell. This implies that a number of gold atoms would be localized in the interdendrimer space, as schematically depicted (Scheme 2). Accordingly, polycrystalline gold nucleates inside the dendrimer molecules that serve as nanoreactor and framework for the future nanocomposites. The gold atoms remaining outside the dendrimers may accumulate and crystallize leading to pure gold crystalline nanoparticles or can contribute to the growth phase of the gold-dendrimer nanocomposites. Another process that can be anticipated is the ripening of the nanocomposites. Nevertheless, it is of paramount importance to state here that the use of gold excess would imply that the stabilization of the nanocomposites by dendrimer molecules is not stoichiometrically favored in the studied cases.

The real situation is even more complex since dendrimers, as mentioned above, are able to interact physically and they do form clusters in aqueous solutions. On the other hand, a higher number of gold atoms are believed to be generated outside the dendrimer nanoreactors, since the internal cages are not large enough to host 254 Au^0^ as stoichiometrically required. Accordingly, the stoichiometric prediction of gold distribution presented in Scheme 2 is just theoretical; the mechanisms governing the nanocomposites formation are extremely complicated and the dendrimer concentration indeed plays an important role. Further in this work, we will present the very interesting experimental data we recorded relative to these aspects.

The reaction mixtures were prepared as previously described. UV-Vis, TEM, and HR-TEM analyses have been performed.

A comparison between the UV-Vis spectra revealed in a first step that the induction time until colloidal gold specific SPR signal was observed is strongly affected by the concentration of PAMAM-SA-G6. Accordingly, when the ratio of Au(III) to tertiary amine is 10 : 1, an SPR signal specific to gold nanoparticles has been detected only after 30 minutes since the moment the reacting species have been put into contact. When increasing ten times the amount of dendrimer (meaning 1 Au(III): 1 tertiary amine), the reduction of gold was extremely fast and the SPR signal was visible in less than 1 minute after the solutions have been mixed. These behaviors are supported by well-known mechanisms and phenomena [12] and have important consequences.

**Figure 6:**
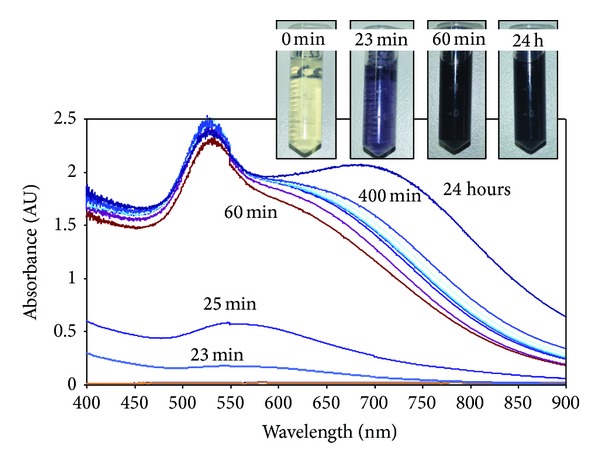
Gold-dendrimers Au-G6-2.5 formation and evolution over 24 hours as recorded by UV-VIS measurements; absorbance was recorded at moment 0, 23 minutes, 25 minutes, 60 minutes, 120 minutes, 205 minutes, 235 minutes, 252 minutes, 300 minutes, 400 minutes, and 24 hours after synthesis, respectively.

To better understand the formation of gold-dendrimer nanocomposites in the presence of PAMAM-SA-G6 molecules, the syntheses were repeated and the evolution of the SPR band has been monitored in time.  Figure 6 is suggestive with this respect and it describes the evolution of the SPR signal in time. After 20 minutes since the precursor and the dendrimer solutions have been mixed, a low shoulder-like signal appeared at 552 nm proving that nanoparticles already started to be formed in the reaction mixture. Only 5 minutes later, a neat but still broad peak was visible at 552 nm. Within the next 35 minutes, the solution turns dark red and a hypsochromic effect from 552 nm to 525 nm is noticed for the SPR signal. This is a typical behaviour associated with potential agglomeration phenomena. Furthermore, a shoulder is noticed above 600 nm; it increases in intensity and presents a bathochromic effect during 24 hours. This behaviour states the formation of larger aggregates. Such phenomena have been noticed for each of the Au-G6 nanocomposites.

**Figure 7:**
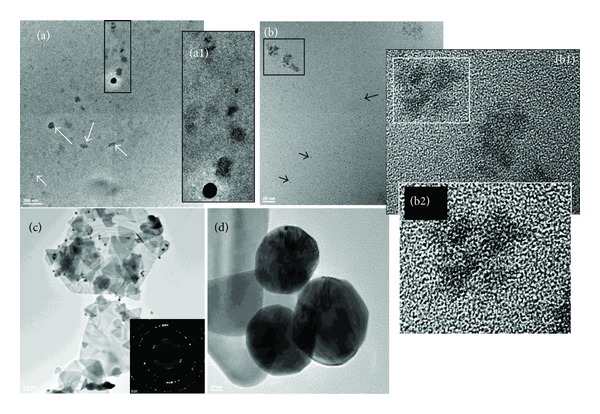
TEM and HR-TEM micrographs characteristic for Au-G6-10 at 60 minutes after preparation ((a), (b)) and at 48 hours after preparation ((c), (d)). The general appearance of the sample after 60 minutes contains two types of areas: (a) few spheroidal particles accompanied by numerous metal agglomeration inside clusters of dendrimers (as indicated by white arrows) and (b) preorganization areas with limited number of metal atoms inside dendrimer cages and metal accumulations outside dendrimers (as shown by black arrows). (a1) Detailed view of the marked area from image (a), showing the metal domains accumulated in one dendrimer molecule. (b1) Detailed view of the marked area from panel (b). (b2) Zoomed area from image (b1). (c)-(d) After 48 hours, two types of crystalline phases exist: spheroidal particles and crystalline structures developed through unhindered growth. Inset represents SAED image characteristic for gold (fcc).

The samples prepared with the lowest dendrimer content presented a typical morphology that is totally different when compared to the other nanocomposites obtained using higher dendrimer concentrations.  Figure 7 contains a selection of TEM micrographs representative for the development of Au-G6-10. 60 minutes after the reagents were mixed, different types of structures are observed: few spherical nanoparticles, numerous areas of metal agglomeration inside clusters of dendrimers (as indicated by white arrows in Figure 7(a)), preorganization areas with limited number of metal atoms inside dendrimer cages, and metal accumulations outside dendrimers (as shown by black arrows in Figure 7(b)). When low amount of gold preorganizes inside dendrimer molecules, before even crystalline domains are formed, spectacular images have been recorded in HR-TEM; the metal domains have been visualized as the negative of the dendrimer host, with four main branches deriving from the central core and higher branching at the periphery of the structure (Figures 7(b1) and 7(b2)). A similar behavior has been described in [12]. Remarkably, these images are obtained at high resolution offering a nice visualization of the intramolecular capturing of gold by PAMAM-SA-G6. Furthermore, to the best of our knowledge, it is the first time when the accumulation of gold in the intermolecular space described by PAMAM-SA dendrimers is visualized. When correlating these findings with the above described prediction with respect to gold distribution inside the dendrimer nanoreactors and in the intermolecular space, we observe that they are in perfect agreement. Furthermore, this corresponds to the observations made by visual inspection and UV-Vis spectrometric results. Interestingly, at this ratio, between gold and the tertiary amines from PAMAM-SA-G6, the gold from the intermolecular sectors forms thin irregular polycrystalline structures, while the intermolecular gold concentrates and generates spheroid nanoparticles (Figures 7(c) and 7(d)). The freely grown gold crystalline phase (developed as triangular or even ribbon-like structures) quantitatively dominates the system as stoichiometrically predicted. SAED typical rings were characteristic to gold (fcc) crystalline structures.

**Figure 8:**
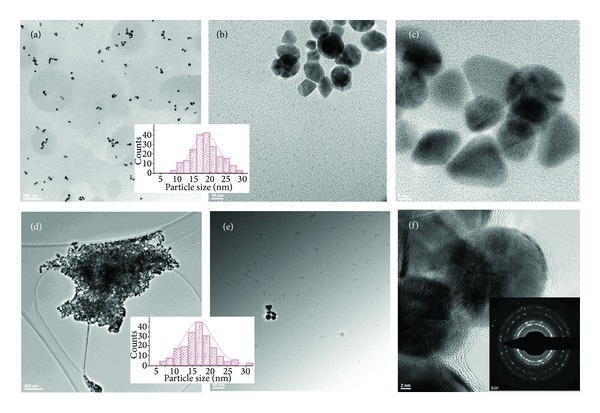
TEM and HR-TEM micrographs characteristic for Au-G6-5 at 60 minutes ((a)–(c)) and 48 hours ((d)–(f)), respectively, after preparation. (a) General appearance of not-yet agglomerated nanoparticles; (b) spheroid and irregular nanoparticles and gold accumulating in the intermolecular space (black arrows); (c) detailed view of crystalline nanocomposites; (d) nanoparticles forming a cluster after 48 hours after their synthesis; (e) few nanoparticles and low concentrations of metal (zoomed area is indicated in black rectangles); (f) HR-TEM micrograph of polycrystalline spherical nanoparticles, with organic layer at the periphery and SAED as an inset. Size-distribution histograms are shown.

Increasing the dendrimer concentration generally leads to smaller nanoparticles as further presented. Augmenting the amount of dendrimer 2 times lead to Au-G6-5 nanocomposites. This system reacted faster when compared to the previously described. TEM and HR-TEM investigations provided again important information of the organization, size, and morphology inside the system at two times: 60 minutes and 48 hours, respectively. After 60 minutes, numerous nonagglomerated spheroid and irregular nanoparticles are observed (Figures 8(a)–8(c)). They are smaller than Au-G6-10 and they coexist with gold accumulating in the intermolecular space. After 48 hours, the morphology of the nanocomposites did not change; however, the particles present an agglomeration tendency (Figure 8(d)). An intriguing aspect is the existence of low concentrations of metal apparently organized inside dendrimer cages (zoomed area is indicated in black rectangles in Figure 8(e)). The polycrystalline nature of the nanoparticles has been confirmed by HR-TEM and SAED. An organic layer was visible at the periphery of the nanoparticles, suggesting an stabilizing effect of the dendrimer molecules. At this dendrimer concentration, the histograms size distribution did not indicate an important modification of the polydispersity and size of the particle in 48 hours. Au-G6-2.5 nanocomposites have smaller dimensions and presented similar properties with Au-G6-5.

**Figure 9:**
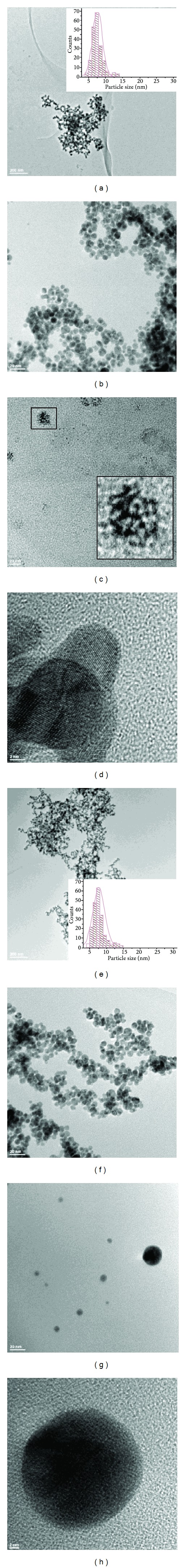
TEM and HR-TEM micrographs of Au-G6-1 nanocomposites: (a)–(d) after 60 minutes and (e)–(h) after 48 hours. (a), (e) Clusters of nanoparticles; (b), (f) chain-like structures of nanoparticles; (c) area with limited number of metal atoms accumulated inside PAMAM-G6 molecules; inset in (c) is a detailed view of the marked area from this image, and it shows gold domains inside one dendrimer molecule; (d) crystalline nanoparticle with adjacent crystalline structures; (g) small metal particles; (h) one nanoparticle. Size-distribution histograms are included as insets in (a), (d).

**Figure 10:**
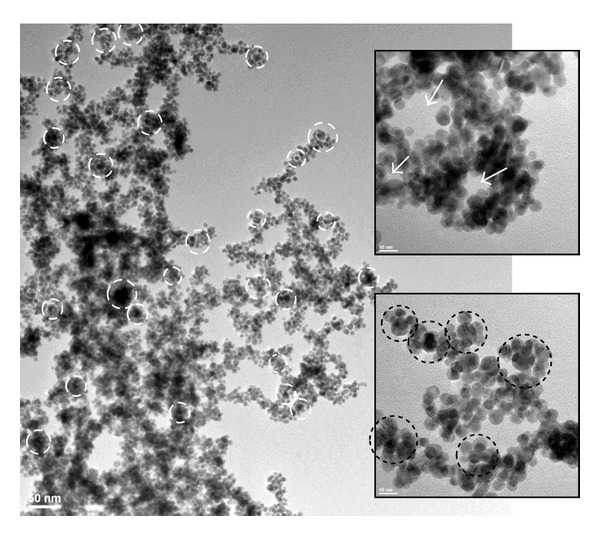
Typical self-assembling of Au-G6-1 nanoparticles, after 48 h, with agglomerations (white and black circles) and interstitial channels (white arrows in upper inset).

Au-G6-1 system offered an even more interesting perspective since these materials are prepared using 1 : 1 gold to tertiary amines ratio. This reaction system initially contained ten times more dendrimer than Au-G6-10. The reduction reaction occurred extremely fast, and the colloidal gold have been obtained in less than 1 minute. Such a high reaction rate and stoichiometry involved a multitude of phenomena occurring simultaneously and/or concurrently. Fast reduction is known to be accompanied with less size homogeneity. However, our results indicate that even at 60 minutes the polydispersity is quite narrow, with an overall average dimension of 7.569 ± 1.429 nm, with minimum structures of 4.015 nm, mean dimensions of 7.421 nm, and maximum dizes of 13.08 nm. Furthermore, after 48 hours, the overall average size remains approximately the same, namely, 7.71 ± 1.785 nm, with minimum particles of 4.67 nm, mean values of 7.44 nm, and maximum structures of 14.57 nm. These results are not in contradiction with the previous statement with respect to the influence of the reduction rate over the size distribution. We would like to emphasize the paramount importance of the stoichiometry of the reaction. We estimated that an equimolecular ratio between the metal and the reducing species would not enhance stabilization effects of the dendrimer, since these molecules are stoichiometrically available as host for the preorganization of the gold (see Scheme 2(d)). Gold precursor did not have the time to completely preorganize inside the dendrimer cages. Hence, at 60 minutes, the typical Au-G6-1 system is formed by small agglomerated nanoparticles (Figure 9(b)) coexisting with areas of gold atoms accumulating inside the dendrimers (Figure 9(c)). Again, HR-TEM allowed the visualization of limited number of gold atoms inside PAMAM-SA-G6 molecules (inset in Figure 9(c)). At 48 hours, the colloid is no longer stable; its propensity to aggregate is confirmed by the visible precipitation of the sample. Vigorous stirring allows dispersion of large particles. TEM revealed agglomerated nanoparticles with almost the same dimension and narrow size dispersity as at 60 minutes. However, we speculate that ripening processes have been favored; no more gold domains embedded in dendrimers have been identified; most probably they have grown and changed into polycrystalline spherical structures as shown in Figures 9(f)–9(h). Furthermore, a tendency of these nanocomposites to self-assemble has been noticed.  Figure 10 is representative for the tendency of Au-G6-1 to form nanostructured networks of entangled necklace-like nanoparticle chains. Typical arrangements consist in a chain of “satellites-planet-” like clusters formed by smaller particles oriented around one large nanoparticle (white and black circled areas in Figure 10). Such arrangements could further enhance ripening phenomena, but these aspects will be investigated in a future study.

### 3.4. Investigation of the Calcification Potential

We combined PHEMA, a recognized biomineralization-inert organic macromolecular substrate, with a hybrid nanostructured surface consisting in gold-dendrimer nanocomposite presenting a carboxylated functional shell. Soaking in SBF1.5x was used to estimate the calcification ability of the developed nanosurface. At the end of the calcification test, FTIR spectra were recorded and compared with a positive control consisting in nano-HA (Sigma Aldrich) and with a negative control consisting in the spectrum of the samples prior to incubation. The calcification of the materials was confirmed through new strong intensity peaks assigned to PO_4_^3−^ characteristic vibrations, visible at about 1030 cm^−1^. In nano-HA, the PO_4_^3−^ band is identified as a narrow peak at 1017 cm^−1^. This spectral modification is considered as the main FTIR feature confirming the biomineralization potential of Au-G6-1-treated PHEMA.

**Figure 11:**
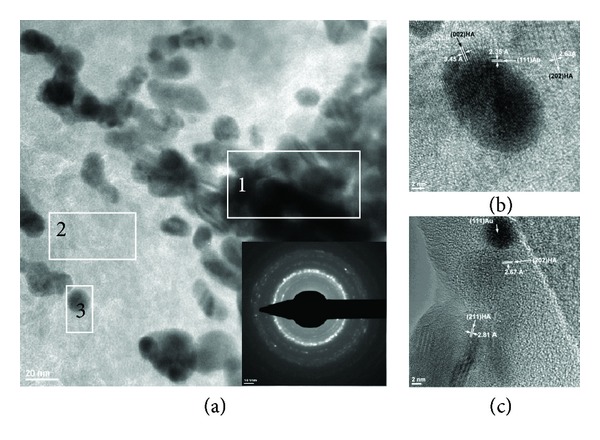
TEM micrographs providing morphostructural evidences on the formation of HA on the Au-G6-1 functionalized PHEMA surface; HA nanocrystals are formed around agglomerations of gold-dendrimer nanocomposites; (a) agglomerations of nanocomposites (area 1) are embedded in a mass of HA (area 2) formed following the incubation in SBF1.5x, while area 3 presents one nanocomposite particle with HA nanocrystals around and the inset 4 represents the SAED image with crystallographic planes characteristic to Au and HA; (b), (c) HR-TEM images presenting one nanocomposite nanoparticles identified through the typical crystallographic planes (111) surrounded by HA nanocrystals with their characteristic planes (002), (202), and (211).

Next, TEM micrographs confirmed the presence of nanocrystals with typical HA morphology. The layers removed from the analysed surfaces indicated the presence of a mass of nanometric HA embedding the composite nanoparticles (Figure 11). Morphological and microstructural details are displayed in Figure 11. HA appears as elongated nanoparticles. The microanalysis indicated crystallographic planes typical to gold and HA as it can be noticed in Figures 11(b) and 11(c). These results are considered promising and justify further studies on the potential of Au-G6 nanocomposite to induce biomimetic mineralization.

## 4. Conclusions

This study demonstrated that gold-dendrimer nanocomposites can be easily generated by a colloidal chemistry approach based on the use, for the first time, of PAMAM-SA dendrimers with dodecanediamine core in the absence of additional reducing agents or other stimulating factors such as light, temperature, or microwaves. We have demonstrated that a rational control of the size, shape, assembling properties, and stability of the nanocomposites can be realized by adjustment of the stoichiometric parameters and appropriate selection of the dendrimer type. Furthermore, nanostructured networks or coatings consisting in entangled chains of self-assembled nanoparticles can be produced using the so-formed nanoparticles. Impressively, the organization of the metal inside the dendrimer host has been visualized by HR-TEM. The influence of the dendrimer architecture and functionality on the structural and morphological features of the resulting nanocomposites has been established. Its time-dependent evolution has been described.

Furthermore, the potential of Au-G6-1 nanocomposites to induce HA formation was also proved through an accelerated calcification test using SBF 1.5x. Further work will be devoted to the in-depth investigation of the biomineralization potential of such Au-PAMAM-SA polycrystalline nanocomposites for bone regeneration applications.
